# Plasma amino acids profile in first-episode psychosis, unaffected siblings and community-based controls

**DOI:** 10.1038/s41598-020-78559-w

**Published:** 2020-12-08

**Authors:** Camila Marcelino Loureiro, Daiane Leite da Roza, Fabiana Corsi-Zuelli, Rosana Shuhama, Helene Aparecida Fachim, Lívia Maria Cordeiro Simões-Ambrosio, Rafael Deminice, Alceu Afonso Jordão, Paulo Rossi Menezes, Cristina Marta Del-Ben, Paulo Louzada-Junior

**Affiliations:** 1 Division of Clinical Immunology, Department of Internal Medicine, Ribeirão Preto Medical School, University of São Paulo, Avenida Bandeirantes, 3900, Monte Alegre, Ribeirão Preto, CEP: 14049-900 Brazil; 2 Population Mental Health Research Centre, São Paulo, Brazil; 3 Center for Research in Inflammatory Diseases, Ribeirão Preto Medical School, University of São Paulo, Ribeirão Preto, Brazil; 4 Division of Psychiatry, Department of Neurosciences and Behaviour, Ribeirão Preto Medical School, University of São Paulo, Ribeirão Preto, Brazil; 5 Department of Endocrinology and Metabolism, Salford Royal Foundation Trust, Salford, UK; 6 Department of Physical Education, State University of Londrina, Londrina, Brazil; 7 Division of Nutrition and Metabolism, Department of Health Sciences, Ribeirão Preto Medical School, University of São Paulo, Ribeirão Preto, Brazil; 8 Department of Preventive Medicine, Faculty of Medicine, University of São Paulo, São Paulo, Brazil

**Keywords:** Molecular neuroscience, Biomarkers, Psychosis, Schizophrenia

## Abstract

Investigations of plasma amino acids in early psychosis and their unaffected siblings are rare. We measured plasma amino acids involved in the co-activation of dopaminergic, GABAergic, glutamatergic, and serotoninergic neurotransmitters in first-episode psychosis (FEP) patients (n = 166), unaffected siblings (n = 76), and community-based controls (n = 166) included in a cross-sectional study. Plasma levels of glutamic acid (GLU), glutamine, glycine, proline (PRO), tryptophan (TRP), tyrosine, serine and GABA were quantified by gas-chromatography-mass spectrometry. We used the generalized linear model adjusted by sex, age, and body mass index for group comparison and paired t-test for FEP-Sibling pairs. FEP had reduced GABA plasma levels compared to siblings and controls (p < 0.05 for both). Siblings had lower GLU, Glx and PRO (p < 0.05 for all) but increased TRP compared to patients and controls (p < 0.05 for both). FEP patients with longer duration of pharmacological treatment and medicated only with antipsychotics had increased GLU compared to FEP with shorter periods, or with those treated with a combination of medications (p < 0.05 for both). Finally, FEP patients treated only with antipsychotics presented higher Glx compared to those with mixed medications (p = 0.026). Our study suggests that FEP have low a GABA plasma profile. Unaffected siblings may be a possible risk group for metabolic abnormalities.

## Introduction

Schizophrenia and other psychosis have a multi-factorial biological background^1,2^. Among the different mediating mechanisms, evidence indicates that abnormal amino acid levels, which underlie changes in the metabolic profile, are correlated with psychosis^3,4^.

Twenty-one intracellular alpha-amino acids are present in the human proteins. Twelve amino acids, named non-essential, are synthesized by molecules provided by the organism and attend the cells necessity, such as glutamic acid (GLU), glutamine (GLN), glycine (GLY), proline (PRO), serine (SER), and tyrosine (TYR); the remaining, which are known as essential amino acids, cannot be synthesized in the body and therefore are supplied by the diet intake, such as tryptophan (TRP)^5,6^. Studies have identified that both nonessential and essential amino acids play an important role in energy metabolism^7,8^, including being the main precursors of neurotransmitters^9^, such as dopamine, glutamate, gamma Amino-n-butyric acid (GABA), and serotonin.

Abnormal levels of amino acids involved in the co-activation of dopaminergic, GABAergic, glutamatergic, and serotoninergic neurotransmitters in the brain of patients with psychosis are well-described findings^10,11^. Nevertheless, these investigations mainly rely on the *post-mortem* brains of chronic schizophrenia patients. This constitutes an important limitation, given the long exposure to pharmacological treatment^12^ and disease comorbidities (diabetes mellitus type 2, dyslipidemia, obesity)^13,14^, which are well-known conditions associated with metabolic profile changes in these patients^15,16^. For these reasons, characterizing the profile of amino acids in the peripheral blood of patients in their first-episode psychosis (FEP) can be a reasonable strategy to overcome these caveats, facilitating the comprehension of the pathophysiological processes of psychosis^17^ and the tailoring of preventive strategies.

In fact, the identification of molecular markers in the peripheral blood of FEP patients is a promising strategy in early intervention services, since it may configure future biomarkers of prognosis and treatment response with less association with confounding factors^18,19^. Even though studies investigating the metabolomic profile of drug-naïve and medicated psychotic patients have associated the deregulated peripheral blood amino-acids levels with the onset and course of the disease^20–22^, all these studies were conducted in small samples of early-onset psychosis, and the different methodologies employed do not permit the generalization of the findings. Furthermore, no studies have yet investigated amino-acid dysregulation in unaffected siblings of FEP patients. The inclusion of individuals that share a common genetic- and environmental background with psychotic patients can facilitate the understanding of the role of familial liability to psychosis.

In this study, we aimed to characterize the profile of amino acids related to the: (i) glutamatergic [glutamic acid (GLU), glutamine (GLN), glycine (GLY), glutamic acid + glutamine (Glx), GLN/GLU ratio, proline (PRO), serine (SER)]; (ii) dopaminergic [tyrosine (TYR)]; (iii) serotoninergic [tryptophan (TRP)], and (iv) GABAergic systems [y-Amino-n-butyric acid (GABA)] in the plasma of FEP patients, non-psychotic siblings and community-based controls as possible biomarkers for early-onset and familial risk for psychosis and to explore the role of antipsychotics in the amino acids profiles.

We selected the aforementioned amino acids for the following reasons: (i) serine and glycine are co-agonists of N-methyl-d-aspartate receptor (NMDAR)^23,24^; glutamine is a non-essential amino acid and the precursor of glutamic acid^25^; proline is a multifunctional amino acid that can modulate the function of glutamic acid decarboxylase^26^ and the glutamic acid itself; (ii) regarding the dopaminergic system, we explored the tyrosine amino acid, which is the precursor of dopamine^27^; (iii) we also investigated the amino acid tryptophan, which is the precursor of serotonin^28^; and finally (iv) we evaluated the GABAergic system, by measuring the GABA plasma levels.

The first hypothesis of the study was that amino acid plasma levels related to the dopaminergic system would be increased, whereas those associated with the glutamatergic, GABAergic, and serotoninergic systems would be decreased in FEP patients compared with non-psychotic siblings and matched community-based controls. The second hypothesis was that these abnormal amino acid plasma levels would be more prominent in subgroups of patients with longer exposure to pharmacological treatment. Finally, the third hypothesis was that siblings would present a profile congruent to an intermediate group between FEP patients and controls.

## Methods

### Subjects

This case-sibling-control study is part of an incidence study of mental disorders with psychotic symptoms named STREAM (Schizophrenia and Other Psychoses Translational Research: Environment and Molecular Biology) conducted in the Ribeirão Preto catchment area, Brazil, between 1st of April 2012 and 31st of March 2015^29^. This Brazilian incidence study is part of the consortium European Network of National Schizophrenia Networks Studying Gene-Environment Interactions (EU-GEI)^30,31^.

We classified FEP patients as individuals who contacted mental health services due to their first manifestation of psychosis and who were treated with antipsychotic medications for the first time. One hundred and sixty-six FEP patients, aged between 16 and 64 years old, took part in the study. We included all FEP patients during the study period who were diagnosed with the spectrum of schizophrenia, such as brief psychotic disorder, schizophreniform disorder, schizophrenia, delusional disorder, and schizoaffective disorder; and affective disorders, such as psychotic bipolar disorder and major depressive disorder with psychotic features.

After the inclusion of patients in the study, we asked their permission also to include their non-affected siblings from which 76 accepted to participate. Furthermore, we recruited 166 age- and sex- matched community-based controls who agreed to take part in the blood collection, as detailed described before^32^. The community-based controls were recruited based on the census tracts, defined by the Brazilian census bureau of representative municipalities of the catchment area, stratified by sex and age of the population at risk^32^.

We included siblings and controls residing at the same catchment area of FEP patients, who had not presented psychotic symptoms lifelong confirmed using a standard diagnostic tool^33,34^. Biological siblings were included considering the genetic and environmental factors shared with their peers, as supported by our recent findings^35,36^.

All participants were given a diagnosis according to the Diagnostic and Statistical Manual of Mental Disorders, Fourth Edition, Text Revision (DSM-IV-TR), assessed by the Structured Clinical Interview for the DSM-IV, clinical version (SCID-CV)^33,34^, applied by trained mental health professionals supervised by senior psychiatrists. Moreover, we evaluated the medical records and the report of family members. The exclusion of psychotic symptoms in siblings and controls was also performed using the SCID-CV.

Exclusion criteria for patients were psychotic symptoms due to another medical or neurological illness, or drug intoxication. Additional exclusion criteria for the siblings and controls were a current or previous history of psychotic disorders.

For all participants, an extensive clinical and sociodemographic characterization was performed, as described previously^36^. For patients, the severity of psychiatric symptoms was assessed using the Brief Psychiatric Rating Scale (BPRS)^37,38^ and the duration of untreated psychosis (DUP) was evaluated using the Nottingham Onset Schedule^39^.

The power analysis based on our previous NMDAR proteins study^32^ indicates that the sample size (166 FEP patients and 166 controls) was adequate to provide a power of 90% (to detect a mean difference of 30% in the variance of the outcome variable) at a two-sided 0.05 significance level (Stata Corp).

Our study was approved by the Clinical Hospital, Ribeirão Preto Medical School, University of São Paulo Ethic committee from Brazil, and all participants (FEP patients and/or their legal guardians, community controls) provided written informed consent for participation in the study (Process number 12606/2012). Parents or a first-degree relative were often considered by the clinician investigators when FEP patients showed reduced capacity to determine data or participate in the study.

We guarantee that all methods were carried out in accordance with relevant guidelines and regulations.

### Plasma samples

All blood samples were collected as closer as possible to the date of FEP patients’ inclusion in the study. Peripheral blood was drawn by venous puncture from all subjects during the day and collected into 4 mL EDTA-coated tubes. We then centrifuged the blood (3500 × *g* for 10 min at 4 °C), and the plasma was transferred to 1.5 mL *eppendorf* tubes and stored at − 80 °C until undergoing gas chromatography analysis. The details of the blood sample collection were previously described^32^.

### Amino acids profile

We used 100 µL from the obtained plasma samples for amino acid quantification using the gas chromatography mass spectrometry technology (GC–MS), as recommended by the most standardized protocol^40^.

The amino acid analysis procedure of EZ:Faast Amino Acid Analysis (EZ:FAAST, PHENOMENEX, CA, USA) consists of a solid phase extraction followed by a by-pass and a liquid extraction. Chromatographic analyses were performed in a GC–MS QP2010 Ultra (SHIMADZU, KYOTO, JAPAN). The determination of amino acids plasma levels involved an initial pre-treatment of the samples with dithiothreitol to release protein-bound these molecules.

One standard amino acid, namely GABA, had its EZ:Faast Kit purchased separately from Sigma-Aldrich (Denmark A/S, DK) at the highest available purity. The standard solution was prepared separately to obtain a curve for GABA: 5–260 nmol/mL to an appropriate volume of milliQ-water. Amino acids plasma samples with high concentrations were diluted until these concentrations reached the range of the calibration curve.

Helium at a flow rate of 1 mL/min was used as the carrier gas. The injector and detector temperatures were maintained at 280 °C and 250 °C, respectively. The column temperature program was initialized at 80 °C and held for two minutes, and then increased to 320 °C at a ramp rate of 20 °C/min. The mass spectrometer was operated in electron ionization mode, and SIM modes were applied to detect and quantify these amino acids. The total run time was 15 min.

A calibration curve at concentrations of 25, 50, 100, and 200 ng/mL was prepared by fortifying blank blood with corresponding analytical working solutions. The linearity of the method was investigated by evaluating the coefficient of determination and was achieved with a minimal of 0.99.

### Statistical analysis

Data were analyzed using SPSS version 20.0 for Windows (SPSS Inc) and Statistical Analysis System (SAS/STAT) software version 9.4^41^. We described the groups’ raw data as mean, standard deviation (SD) and frequencies. Before the analysis, the amino acid plasma levels were transformed to a logarithmic scale to improve the normality and reduce the heterogeneity of variance, as often observed in published metabolic studies^42–44^.

We analyzed the sociodemographic and clinical variables among the groups (FEP patients, siblings, and controls) using Pearson’s chi-square and one-way ANOVA with Bonferroni correction. Controls were sex- and age-matched to the FEP patients.

Firstly, we investigated plasma amino acids differences among the three groups by using the generalized linear model (GLM)^45^, including the values of each amino acid as the outcome variables and groups as fixed factor, while adjusting for the effects of age, sex, and body mass index (BMI). Next, using the GLM model, we analyzed differences between amino acid plasma levels within the FEP group and their association with clinical variables while adjusting for the effects of age, sex, and BMI. Multiple comparisons were performed by orthogonal contrasts, and for each of the GLM models performed, the normality of the residuals was checked using normal probability plots. We calculated the effect size among the three groups based on ANOVA and we used the effect size index η^2^ for regression models and analysis of variance (η^2^ values, small: 0.01–0.05, medium: 0.06–0.13, large: ≥ 0.14)^46,47^.

Moreover, we performed an additional analysis in a sub-sample of FEP-Siblings correspondent pairs in relation to amino acid plasma levels using paired T-test and considered sex, age, and BMI as covariates. The effect size calculation was based on differences between the means for FEP-Sibling paired samples, using the effect size of Cohen’s d (d values, small: 0.20–0.49, medium: 0.50–0.79; large: ≥ 0.80)^46,47^. As a common language for the effect size statistics (variance η^2^; three groups) and means (d; FEP-Sibling pairs), we used an equivalence based on z-score probabilities^48^.

For all analyses, BMI data was recorded as a categorical variable according to the World Health Organization classification^49^, and for DUP and duration of treatment we used the same categorization as published in our previous study^32^.

Finally, in the FEP group, we tested for potential associations among the amino acids analyzed and some patients’ clinical features using GLM models. We considered Glx as a combination of GLU and GLN, as well as the GLN/GLU ratio.

In this study, no missing data were found for any participant in relation to each variable of interest.

Values of p < 0.05 were considered significant for all the analyses.

## Results

### Sociodemographic features of the sample

In this study, 166 FEP patients, 76 biological siblings and 166 controls matched for sex and age, accepted the invitation for peripheral blood collection, and the respective samples were included in all amino acids analysis. Our results (Table 1) showed that the sibling group was composed of a higher percentage of women when compared to both FEP patients and controls (p < 0.001). Moreover, FEP patients had a lower education level (less than 9 years of schooling), and reported less relationship bound in relation to siblings and controls (p < 0.001 for both). The percentage of FEP patients who were not currently working was higher than siblings and controls (p = 0.015).

**Table 1:** Sociodemographic and clinical variables from FEP patients, non-psychotic siblings and matched community-based controls.

	Control group (n = 166)	Sibling group (n = 76)	FEP patients (n = 166)	Test	p-value^c,d^
**Sociodemographic data: n (%), mean (SD)**					
Age, years	31.4 (12.0)	31.5 (11.0)	30.3 (12.2)	F_(2,407)_ = 0.395	0.674
Sex, male	106 (63.9)^b^	23 (30.3)^a^	106 (64.0)^b^	χ^2^ = 28.574	** < 0.001**
Education, ≤ 9 years	40 (24.1)^b^	23 (30.3)^b^	95 (57.2)^a^	χ^2^ = 41.217	** < 0.001**
Ethnicity, white	113 (68.1)^a^	39 (51.3)^b^	81 (50.0)^b^	χ^2^ = 12.519	**0.002**
Marital status, single	83 (50.0)^b^	32 (42.1)^b^	123 (74.1)^a^	χ^2^ = 29.947	** < 0.001**
Occupation, currently not working	67 (40.4)^b^	24 (31.6)^b^	84 (50.6)^a^	χ^2^ = 8.434	**0.015**
**Clinical data: mean (SD)**					
Body mass index, kg/m^2^	26.5 (5.5)^a^	24.9 (4.9)^a,b^	24.7 (5.0)^b^	F_(2,407)_ = 4.825	**0.013**
Abdominal circumference, cm	89.1 (15.1)^a^	82.9 (14.4)^b^	86.1 (13.3)^a,b^	F_(2,331)_ = 4.286	**0.016**
**Use of psychoactive substances: n (%)**^**e**^				χ^2^ = 87.107	** < 0.001**
Cannabis only	15 (9.0)	4 (5.3)	9 (5.4)		
Cannabis combined with others	20 (12.0)^b^	2 (2.6)^b^	76 (45.8)^a^		
Other substances	11 (6.6)	0 (0.0)	12 (7.2)		
None	120 (72.4)	70 (92.1)	69 (41.6)		
Cigarette smoking	31 (18.7)^b^	13 (17.1)^b^	65 (39.2)^a^	χ^2^ = 22.191	** < 0.001**
**Only FEP patients: mean (SD)**					
DUP, weeks			59.9 (173.6)		
Duration of treatment, weeks			32.5 (42.1)		
BPRS total score			9.1 (6.9)		
Median of psychosis until blood collection			35.5		
Median of treatment until blood collection			12.5		

*FEP* first-episode psychosis, *SD* standard deviation, *DUP* Duration untreated psychosis, *BPRS* brief psychiatric rating score.

Bold: p ≤ 0.05.

^a,b^Means followed by the same letter did not differ statistically from each group in the table. Different subscript letters represent the significant differences between groups.

^c^One-way ANOVA with Bonferroni correction.

^d^Chi-square test.

^e^Use of psychoactive substances (current or lifetime): Cannabis; alcohol; cocaine/crack; inhalants; amphetamine.

Regarding ethnicity, taking as a proxy the self-declared skin color, the percentage of white subjects was higher in controls than FEP patients and siblings (p = 0.002). Controls presented higher BMI than FEP patients (p = 0.013) and higher abdominal circumference measurements than siblings (p = 0.016). For life-time psychoactive substance use, FEP patients reported more cannabis use in combination with other illegal substances and more tobacco smoking than siblings and controls (p < 0.001 for both).

The median of the duration of psychosis until the blood collection was 35.5 weeks, while the median of pharmacological treatment in relation to the blood collection was 12.5 weeks. The majority of the patients were followed in an early intervention service, with a good response to the initial treatment (median BPRS = 9.1). More details of other sociodemographic and clinical variables were described previously^32^.

### Amino acid plasma levels: group comparison

**Figure 1:**
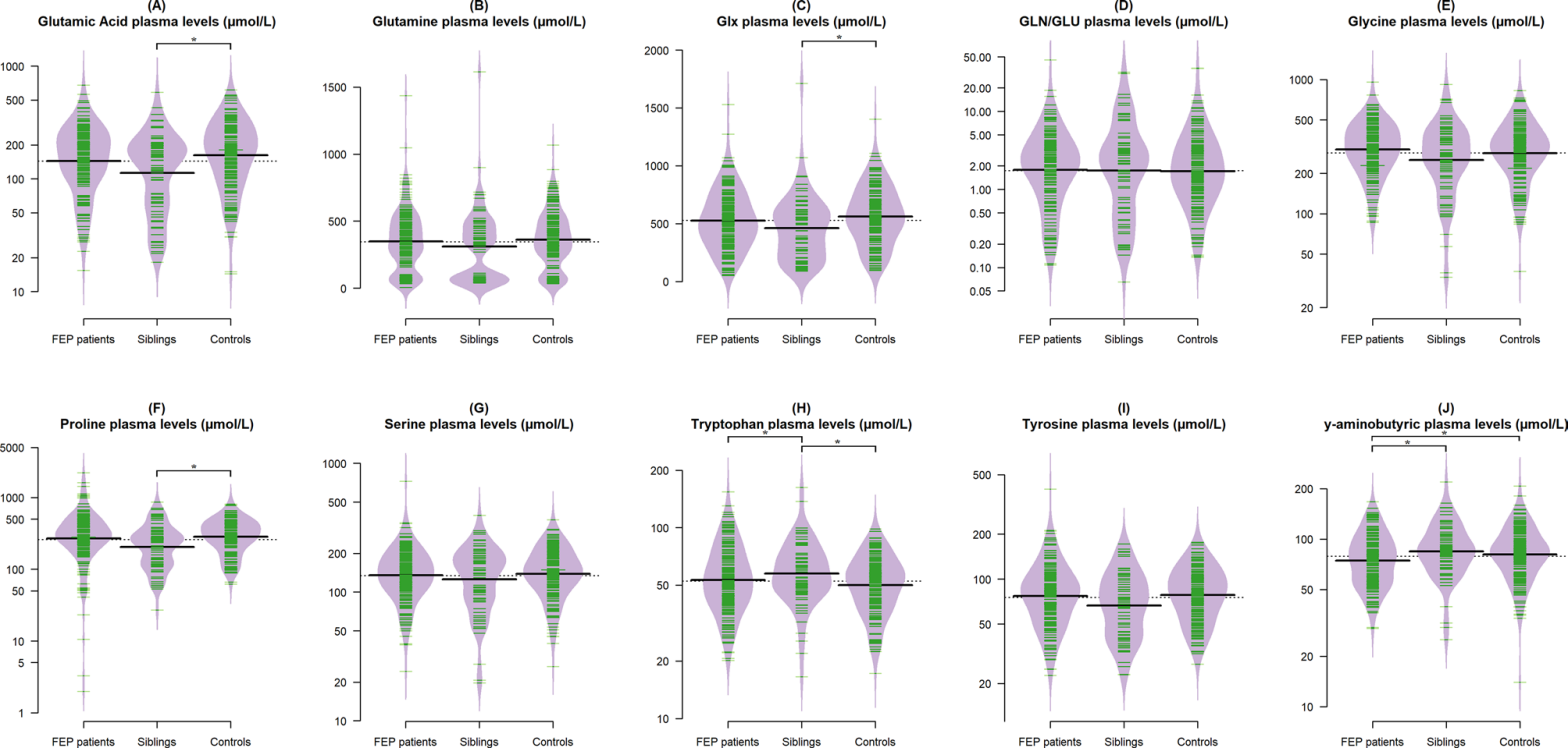
Beanplot of glutamic acid (**A**), glutamine (**B**), Glx (**C**), GLN/GLU ratio (**D**), glycine (**E**), proline (**F**), serine (**G**), tryptophan (**H**), tyrosine (**I**), and ÿ-aminobutyric (**J**) plasma levels (µmol/L, raw values) of FEP, siblings and controls (n = 408). *Group comparison; p < 0.05. The data was analyzed by the generalized linear model adjusted by sex, age and BMI.

The Fig. 1A–J and Table 2 describe the (A) GLU, (B) GLN, (C) Glx, (D) GLN/GLU ratio, (E) GLY, (F) PRO, (G) SER, (H) TRP, (I) TYR, and (J) GABA plasma levels of FEP patients, non-affected siblings, and community-based controls.

**Table 2:** The effect sizes and the common language effect size statistic correspondent between FEP-sibling-controls in relation to amino acid plasma profile.

Amino acids	FEP	Siblings	Controls	FEP-sibling-controls			
	Mean (SD)			F	p-value	η^2^ (95% CI)	CL (%)*
GLU	178.03 (110.80)	149.20 (104.74)	200.83 (123.05)	3.43	**0.002**	0.06 (0.01, 0.12)	60.0
GLN	349.22 (228.50)	310.74 (268.68)	360.99 (210.81)	1.45	0.062	0.03 (0.01, 0.06)	51.0
GLY	331.99 (141.39)	298.61 (165.95)	316.68 (143.92)	2.64	0.198	0.05 (0.01, 0.09)	59.0
Glx	527.25 (253.65)	459.94 (275.84)	561.83 (249.79)	2.36	**0.004**	0.05 (0.01, 0.08)	59.0
GLN/GLU	3.10 (4.38)	3.97 (5.82)	3.04 (4.54)	0.34	0.953	0.01 (0.00, 0.01)	54.0
PRO	365.29 (298.61)	255.47 (170.42)	324.93 (158.56)	3.20	**0.005**	0.06 (0.02, 0.10)	60.0
SER	150.30 (76.33)	145.20 (72.28)	152.05 (62.42)	3.03	0.317	0.06 (0.02, 0.10)	60.0
TRP	57.70 (23.75)	61.42 (23.54)	52.81 (17.11)	3.18	**0.022**	0.06 (0.02, 0.10)	60.0
TYR	86.41 (45.81)	74.80 (36.05)	85.09 (33.72)	2.62	0.214	0.05 (0.02, 0.08)	59.0
GABA	79.29 (28.30)	90.15 (32.78)	86.23 (29.84)	1.70	**0.023**	0.04 (0.01, 0.06)	57.0

*η*^*2*^ eta-squared (small: 0.01–0.05, medium: 0.06–0.13, large: ≥ 0.14), *CI* confidence interval, *CL common language effect size statistic.

Bold: p ≤ 0.05.

FEP patients showed reduced GABA plasma levels in comparison to both siblings (p = 0.006) and controls (p = 0.048). In addition, siblings had lower plasma levels of GLU (p = 0.006), Glx (p = 0.005) and PRO (p = 0.021), but increased TRP plasma levels in comparison to patients (p = 0.021) and controls (p < 0.001).

Finally, we did not find significant differences between the groups in relation to GLN (p = 0.062), GLN/GLU ratio (p = 0.953), GLY (p = 0.198), SER (p = 0.317), and TYR (p = 0.214).

In the secondary analysis of FEP-Siblings pairs comparison, we did not find any statistical significant differences in any amino acids profile (GLU: p = 0.852; GLN: p = 0.556; GLY: p = 0.773; Glx: p = 0.388; GLN/GLU: p = 0.607; PRO: p = 0.619; SER: p = 0.561; TRP: p = 0.091; TYR: p = 0.384; GABA: p = 0.124). Detailed information is described in Supplementary Table S1.

### Associations between amino acid plasma levels and socio-demographic and clinical characteristics in FEP patients

We did not find significant differences in amino acid plasma levels in relation to sex, age, psychoactive substances use, and tobacco smoking. However, FEP patients with BMI between 25.0 to 35.9 kg/m^2^ had higher Glx plasma levels in comparison to FEP patients with BMI between 18.5 to 24.9 kg/m^2^ (p = 0.010) and BMI higher than 31.0 kg/m^2^ (p = 0.006). No other significant associations were observed.

FEP patients with up to 12 weeks of DUP had lower GLU (p = 0.005) and TYR (p = 0.002) plasma levels in comparison to those with more than 13 weeks of DUP [13 to 24 weeks, GLU (p = 0.032) and TYR (p = 0.044); and with DUP higher than 53 or more weeks, TYR (p = 0.009)].

In addition, GLU plasma levels changed in relation to different durations of treatment. FEP patients with up to 11 weeks of pharmacological treatment presented decreased GLU plasma levels in relation to FEP patients with 12 or more weeks (p = 0.009). Moreover, FEP patients treated with antipsychotics only had significantly increased GLU plasma levels when compared to FEP patients treated with a combination of medications (p = 0.010). FEP patients treated only with antipsychotics presented higher Glx plasma levels when compared to FEP patients with antipsychotic associated with other medications (p = 0.026).

Finally, we analyzed the FEP patients according to three categories of antipsychotic treatments: none (n = 9), atypical (n = 74) and typical (n = 83); the use or type of antipsychotics did not change any amino acid plasma concentrations (Table 3).

**Table 3:** Differences between amino acid plasma levels in relation to clinical variables in FEP patients.

Clinical variables	Amino acids plasma levels (µmol/L)^c^									
	GLU	GLN	Glx	GLY	GLN/GLU	PRO	TRP	TYR	SER	GABA
	Mean (SD)									
**Sex**	p = 0.387	p = 0.436	p = 0.387	p = 0.652	p = 0.735	p = 0.693	p = 0.984	p = 0.464	p = 0.794	p = 0.467
Female (n = 60)	164.0 (101.9)	335.0 (228.6)	499.0 (263.6)	318.1 (148.7)	3.0 (3.2)	368.4 (348.8)	57.1 (22.5)	78.9 (33.8)	139.8 (59.6)	75.9 (26.3)
Male (n = 106)	186.0 (115.3)	357.3 (229.1)	543.2 (247.7)	339.8 (137.2)	3.1 (4.9)	363.6 (267.8)	58.1 (24.5)	90.7 (51.1)	156.2 (84.0)	81.2 (29.3)
**Age**	p = 0.967	p = 0.386	p = 0.844	p = 0.072	p = 0.838	p = 0.410	p = 0.766	p = 0.726	p = 0.057	p = 0.896
16 to 24 (n = 66)	176.9 (96.2)	369.4 (226.5)	546.3 (249.8)	362.4 (122.6)	2.9 (2.6)	412.3 (324.6)	56.9 (24.2)	88.6 (37.1)	163.4 (57.5)	79.5 (25.0)
25 to 34 (n = 47)	177.3 (117.4)	355.3 (218.6)	532.7 (243.0)	315.0 (140.8)	3.2 (3.4)	309.3 (207.2)	58.5 (22.8)	85.9 (59.4)	135.0 (50.6)	79.0 (25.7)
35 or more (n = 53)	180.1 (123.2)	318.7 (240.3)	498.8 (269.4)	309.2 (158.5)	3.3 (6.5)	356.4 (327.9)	58.1 (24.4)	84.1 (42.6)	147.5 (107.9)	79.3 (34.3)
**BMI (kg/m**^**n**^**)**	p = 0.233	p = 0.264	**p = 0.001**	p = 0.078	p = 0.437	p = 0.597	p = 0.919	p = 0.942	p = 0.210	p = 0.223
Up to 18.4 (n = 11)	155.1 (66.5)	435.3 (227.4)	548.1 (213.2)	366.2 (153.8)	3.4 (2.9)	303.6 (123.8)	59.4 (22.5)	86.0 (33.1)	153.1 (59.4)	74.7 (23.8)
18.5 to 24.9 (n = 101)	194.9 (119.5)	339.2 (242.1)	594.6 (289.5)	349.8 (143.6)	3.5 (6.4)	385.9 (246.5)	58.1 (21.7)	83.7 (32.7)	151.6 (60.9)	78.3 (24.8)
25.0 to 30.9 (n = 42)	158.4 (91.2)	334.3 (197.8)	451.0 (217.4)	296.9 (130.5)	2.5 (2.2)	382.4 (365.8)	57.4 (26.4)	88.5 (54.5)	156.4 (90.6)	83.7 (31.9)
31 or more (n = 12)	228.0 (159.5)	407.3 (210.8)	648.0 (214.1)	273.5 (122.4)	4.1 (4.6)	262.2 (116.1)	56.7 (19.2)	85.8 (47.5)	117.2 (50.0)	65.2 (18.1)
**DUP (weeks)**	**p = 0.032**	p = 0.673	p = 0.169	p = 0.624	p = 0.404	p = 0.658	p = 0.164	**p = 0.002**	p = 0.356	p = 0.806
Up to 12 (n = 89)	148.7 (92.6)^a^	345.1 (245.7)	493.8 (260.0)	319.7 (141.3)	3.7 (5.6)	342.1 (250.3)	53.9 (21.7)	74.8 (34.3)^a^	141.3 (63.4)	78.0 (30.0)
13 to 24 (n = 28)	223.3 (137.3)^b^	322.0 (187.8)	545.2 (222.2)	351.6 (165.6)	2.2 (2.2)	367.9 (307.1)	62.4 (24.5)	95.1 (34.9)^b^	156.0 (57.1)	78.4 (24.7)
25 to 52 (n = 24)	217.4 (114.4) ^b^	401.8 (232.6)	619.2 (268.6)	344.0 (130.7)	2.7 (2.6)	413.9 (412.3)	63.0 (28.4)	95.8 (40.6)	152.1 (57.9)	80.1 (24.1)
53 or more (n = 25)	194.0 (108.7)	343.8 (206.0)	537.8 (237.9)	342.4 (125.2)	2.2 (1.8)	398.3 (329.8)	60.9 (24.3)	109.0 (77.0)^b^	174.1 (132.2)	84.0 (30.4)
**Duration of treatment (weeks)**	**p = 0.009**	p = 0.801	p = 0.213	p = 0.433	p = 0.599	p = 0.379	p = 0.432	p = 0.191	p = 0.380	p = 0.636
None (n = 9)	171.1 (109.5)	358.4 (165.1)	529.5 (191.9)	367.9 (131.4)	3.0 (2.0)	486.7 (389.0)	59.1 (22.3)	85.7 (44.3)	188.2 (83.6)	89.2 (37.0)
Up to 11 (n = 71)	153.6 (105.6)^a^	338.8 (217.9)	492.4 (260.4)	348.6 (148.8)	3.5 (5.6)	356.0 (304.9)	56.2 (26.0)	79.5 (37.2)	147.8 (56.3)	80.6 (29.6)
12 or more (n = 86)	198.9 (112.1)^b^	356.9 (244.0)	555.8 (252.5)	314.6 (137.8)	2.7 (3.2)	360.2 (284.0)	58.8 (22.1)	92.2 (51.7)	148.4 (88.8)	77.2 (26.2)
**Type of antipsychotic**	p = 0.141	p = 0.100	p = 0.900	p = 0.781	p = 0.990	p = 0.375	p = 0.676	p = 0.161	p = 0.050	p = 0.661
None (n = 9)	172.4 (109.5)	352.7 (165.1)	529.5 (191.9)	354.7 (131.4)	3.0 (2.0)	472.9 (389.0)	58.7 (22.3)	85.7 (44.3)	184.4 (83.6)	89.0 (37.0)
Atypical (n = 74)	199.3 (119.4)	312.1 (232.9)	540.7 (272.0)	337.2 (142.4)	3.1 (2.7)	362.2 (228.5)	59.8 (25.5)	93.8 (53.2)	162.4 (91.4)	80.5 (28.8)
Typical (n = 83)	159.7 (100.5)	381.9 (227.8)	511.9 (240.4)	324.8 (142.5)	3.1 (5.9)	356.4 (341.2)	55.8 (22.4)	79.9 (37.5)	135.8 (55.7)	77.1 (26.9)
**Current treatment**	**p = 0.010**	p = 0.257	**p = 0.031**	p = 0.500	p = 0.440	p = 0.428	p = 0.979	p = 0.489	p = 0.371	p = 0.644
None (n = 9)	171.1 (109.5)	358.4 (165.1)	529.5 (191.9)	367.9 (131.4)	3.0 (2.0)	486.7 (389.0)	59.1 (22.3)	85.7 (44.3)	188.2 (83.6)	89.2 (37.0)
Antipsychotics only (n = 66)	206.4 (111.3)^a^	378.9 (238.7)	585.3 (264.6)	315.6 (111.1)	2.5 (2.5)	365.6 (253.4)	56.3 (19.1)	92.0 (52.3)	143.9 (52.7)	77.6 (25.7)
Antipsychotics and/or others (n = 91)	158.1 (107.2)^b^	326.8 (225.8)	484.9 (244.6)	340.3 (160.6)	3.5 (5.5)	353.1 (319.4)	58.6 (26.9)	82.4 (40.7)	151.2 (88.8)	79.5 (29.3)
**Psychoactive substances**	p = 0.606	p = 0.677	p = 0.940	p = 0.381	p = 0.668	p = 0.438	p = 0.502	p = 0.269	p = 0.770	p = 0.522
None (n = 100)	173.9 (111.6)	347.5 (242.2)	521.4 (261.0)	309.1 (138.9)	3.4 (5.3)	384.1 (348.9)	59.0 (24.4)	80.3 (38.5)	140.0 (65.3)	79.4 (30.1)
Cannabis only (n = 27)	167.6 (107.0)	368.3 (193.6)	535.9 (239.7)	361.0 (124.2)	3.2 (2.4)	388.4 (219.4)	54.1 (21.2)	86.5 (38.0)	166.9 (57.6)	79.5 (27.6)
Cannabis and othersd (n = 20)	205.8 (138.0)	322.7 (208.7)	528.5 (237.4)	355.9 (93.4)	2.3 (2.2)	347.1 (192.5)	56.4 (24.7)	93.4 (38.0)	161.8 (52.2)	84.9 (25.9)
*Others only (n = 19)	185.3 (78.9)	359.0 (233.1)	544.3 (268.4)	385.9 (194.2)	2.4 (2.6)	252.7 (150.4)	57.7 (23.9)	110.9 (81.1)	168.9 (144.1)	72.4 (21.7)
**Use of nicotine**	p = 0.746	p = 0.377	p = 0.701	p = 0.710	p = 0.907	p = 0.148	p = 0.515	p = 0.380	p = 0.382	p = 0.570
Yes (n = 65)	173.0 (96.1)	370.0 (215.8)	543.0 (248.5)	341.7 (128.1)	3.0 (2.5)	317.6 (211.4)	56.9 (23.7)	81.9 (32.9)	147.1 (63.6)	80.9 (25.8)
No (n = 101)	181.3 (119.6)	335.8 (236.4)	517.1 (257.6)	325.8 (149.6)	3.2 (5.3)	396.0 (340.7)	58.2 (23.9)	89.3 (52.4)	152.4 (83.7)	78.3 (29.9)

*GLU* glutamic acid, *GLN* glutamine, *GLY* glycine, *PRO* proline, *TRP* tryptophan, *TYR* tyrosine, *SER* serine, *GABA* y-aminobutyric, *SD* standard deviation, *BMI* Body Mass Index, *DUP* duration of untreated psychosis.

Bold: ≤ 0.05.

^a,b^Superscript letters were used for the mean values in the columns, in which the means followed by different letters differ statistically from each other. Multiple comparisons and orthogonal contrasts performed in the GLM with 5% probability.

^c^Generalized Linear Model adjusted by sex, age and BMI analysed with log transformed.

^d^Alcohol, cocaine/crack and inhalants.

## Discussion

We aimed to characterize the amino acid plasma profile related to the dopaminergic, glutamatergic, serotoninergic, and GABAergic systems in the early stages of psychosis. FEP patients did not differ from community-based controls, except by decreased GABA plasma levels, which was also observed in relation to their unaffected siblings. On the other hand, the non-affected siblings showed significant differences from controls in the plasma levels of amino acids, specifically related to the glutamatergic and serotoninergic systems. Altered amino acids plasma profile in unaffected siblings may be related to familial risk to psychosis since they share a similar genotype and environmental factors with FEP patients.

### Plasma amino acids profile in FEP patients in relation to non-psychotic siblings and community-based controls

Our findings of lower GABA plasma levels in FEP patients compared to non-psychotic siblings and community-based controls are consistent with the literature suggesting decreased GABA in *post-mortem* brain tissues^50,51^, as well as in the plasma of patients with chronic schizophrenia^52,53^. However, a recent study reported higher levels of GABA in drug-naïve schizophrenia patients in comparison to controls^17^. Considering that, in our sample, the decreased GABA was independent of pharmacological treatment; the discrepant results could be justified based on the technique used for the amino acid quantification. Cao et al.^17^ applied the hydrophilic interaction liquid chromatography that has been used for polar metabolites; however, this method has disadvantages when compared to the method used in our study, especially due to its longer retention time drifts and extensive re-balance runs. To overcome that, we used the GC–MS system that has shown a higher capacity of separation, sensitivity and selectivity of amino acids. This methodology has been reported to be useful for metabolomics studies for providing quick screening approaches^54^. To the best of our knowledge, our study is the first that investigated GABA amino acid plasma levels using the GC–MS system in FEP patients.

The GABAergic system seems to play a central role in the neurobiology of schizophrenia and other psychoses, and disturbances in this system are suggested to contribute to core psychotic symptoms^55,56^. The decreased GABA levels in FEP patients is thought to be a secondary dysfunction from the well-known NMDAR hypofunction on inhibitory interneurons in schizophrenia^57,58^, or may also result from the interaction among this neurotransmitter with others, such as those related to the glutamatergic and dopaminergic systems^58,59^. Although speculative, the reduced GABA levels in the peripheral blood could be reflecting the GABA low concentration observed in the brain of FEP patients^60,61^, potentially suggesting concomitant blood alterations in GABA as a biomarker for psychosis^52,62^.

Contrasting our hypothesis, no differences were found in FEP patients compared with controls regarding their GLU, GLN, GLY, Glx, GLN/GLU ratio, SER, TRP, and TYR plasma levels. However, the findings regarding amino acid plasma levels in psychosis are still contradictory. For instance, while some studies did not show differences in GLU serum levels between schizophrenia patients and healthy controls^63^, other recent investigations found higher^20,42^ or lower^64^ GLU serum/plasma levels in psychotic patients.

Moreover, GLY and SER have been investigated as possible clinical markers for schizophrenia, with some studies demonstrating increased GLY and SER plasma levels in patients after antipsychotic treatment^23,65^; no differences in patients medicated with different antipsychotics^3^, or lower GLY and SER plasma levels in patients in comparison to controls^66,67^. A recent study demonstrated high plasma GLN levels in drug-naïve FEP patients, which was negatively correlated with negative symptoms; however no differences in plasma GLN levels were found after treating these patients^68^.

The discrepant findings could be justified by the distinct methodologies used for amino acid measurement, as well as by specificities of the samples, such as the duration of disease, severity of symptoms, duration of treatment and type of antipsychotics, dietary habits, and the amino acid measurement made by in the peripheral access^22^. In our study, we can justify the absence of the differences between FEP patients and controls by some features of our study (the case-sibling-control design, inclusion of affective and non-affective psychosis, not fasting plasma samples, and the heterogeneity in the duration of treatment and psychosis).

Particularly, in our study, treatment with antipsychotics may have brought the amino acid plasma levels closer to the normal range and may be one of the reasons why we did not find differences between FEP patients, siblings, and controls^22^. Corroborating with this explanation, we found that low DUP, short-duration of antipsychotic treatment, and use of antipsychotics combined with other medication were associated with low GLU plasma levels. Low DUP was also associated with reduced TYR plasma levels.

### Plasma amino acids profile in non-psychotic siblings in comparison to community-based controls

Our data showed decreased GLU, Glx and PRO plasma but higher TRP plasma levels in non-psychotic siblings when compared to community-based controls.

We identified three studies investigating the metabolic profile in the brain regions of patients’ siblings using the proton magnetic resonance. One, in agreement with our findings, found lower prefrontal GLU in unaffected twins of schizophrenia patients when compared to controls^69^. In the same direction, a recent study showed lower cortical GLU in first-degree relatives compared with healthy controls, but no differences were observed between the relatives and schizophrenia patients^50^. However, a third study, did not find differences in GLU in adult siblings of schizophrenia patients compared with healthy volunteers, which can be justified by the small sample size and the use of unsegmented metabolite values^70^. Different from our study, these previous studies have not investigated the TRP and PRO plasma levels in unaffected siblings. Taken together, reduced peripheral GLU, Glx, and PRO levels and increased TRP levels may be reflecting imbalances in the amino acids plasma profile related to familial risk to psychosis, which may potentially be considered as an indicator of the biological vulnerability associated with psychosis, since non-affected siblings share a similar genotype and early-life environment with FEP patients.

### Limitations and strengths

Our results should be interpreted with some caution. First, we measured the amino acids at one-time point and did not control for the participants’s diet. We cannot exclude the influence of fasting condition at the time of blood sampling in the amino acid plasma levels, given the fact that some of the amino acids are taken by diet, and that previous evidence demonstrated positive associations between food consumptions and altered profile of amino acids^5,71^. In addition, we do not have data concerning the physical activity of the participants, which can also impact the amino acids profile^72,73^ similar to information about metabolic diseases as dyslipidemia and diabetes mellitus. Third, our patients were not drug-naïve and were heterogeneous regarding the type and duration of pharmacological treatment. Fourth, we were not able to investigate a brain-blood correspondence of the amino acid profile, using methods such as resonance imaging spectroscopy. It is important to highlight that our data is related to plasma amino acids levels, which may not correlated with amino acids levels in the brain. Interestingly, evidence has demonstrated that some human brain amino acids, such as GLU, is correlated to the peripheral pathway in schizophrenia patients^74^. Furthermore, findings have suggested that TRP and TYR peripheral alterations may reflect brain changes due to their transportation by neutral amino acid carrier system^75,76^. Thus, although speculative, the altered amino acid profiles in the peripheral blood might, to some extent, reflect those changes observed in the brain^76^. However, future studies measuring blood and brain amino acid activities are needed to conclude their correlation and potential role as biomarkers in psychosis. Fifth, we did not perform an analysis of amino acids according to FEP heterogeneity. Future studies are necessary to evaluate the amino acid plasma levels considering the FEP patients in different diagnostic categories (affective and non-affective psychoses) since the diagnostic stability in first-episode psychosis is low, considering other clinical diagnoses and compared to controls. Sixth, we were only able to measure the racemic serine (DL-serine) using GC–MS in the peripheral blood, given that the Phenomenex lab Kit does not separate their chiral forms. D-serine that bind to GLY, a co-agonist of NMDAR, has an important role in the NMDAR function as the synaptic plasticity^77^. Finally, the assessment of metabolic diseases relied only on self-reported data collection, which can underestimate the formal clinical diagnoses.

Despite these important limitations, our paper has several strengths. We included FEP patients with a detailed clinical characterization allowing exploration of the association between psychotropic treatment and other clinical variables with the amino acid plasma levels. Furthermore, we included their biological siblings, considered a risk group that shares the same environment and similar genetic profile of the patients, instead of the traditional case–control design; we also included community-based controls representative of the general population. Finally, our study used the GC–MS, a technique that provides a robust profile of amino acid on their molecular level. This approach has often been considered a gold standard tool to explore amino acid profiling in metabolomic studies^78,79^, which had not yet been employed by other psychosis studies. We can also consider the generalizability of our findings as satisfactory. Our metabolite approach offers a low complexity of amino acids data acquisition, with high-sensitivity, fast time and low-cost to screening the metabolite features involved in predefined pathways implicated in the pathophysiology of psychosis.

## Conclusions

Our study suggests that FEP patients may be characterized by a reduced GABA plasma profile comparedto community-based controls and their unaffected siblings. Furthermore, we were able to show the influence of patients’ pharmacological treatment in the GLU plasma profile. Our results also suggest that metabolic abnormalities, especially those associated with decreased GLU, Glx and PRO, are not restricted to psychosis, bringing the unaffected siblings as a possible risk group for metabolic abnormalities. Finally, the GC–MS technique should be considered a useful tool for the screening of peripheral amino acids, contributing to the comprehension of the pathophysiological processes in psychosis and their healthy first-relatives. Altogether, the peripheral blood alterations seen herein might reflect an imbalance of amino acids in psychosis. Further studies, especially metabolomics, are needed to confirm our findings and improve the understanding of psychosis pathogenesis.

## Supplementary Information

Supplementary Information.
