# Supplementary material for: Plasma amino acids profile in first-episode psychosis, unaffected siblings and community-based controls
Source: Sci Rep. 2020 Dec 8;10:21423. doi: 10.1038/s41598-020-78559-w (PMC7722891; doi:10.1038/s41598-020-78559-w)
Supplement: Supplementary file 1 — Supplementary Information. [file 41598_2020_78559_MOESM1_ESM.docx]

**Table S1.** Comparisons between FEP-Sibling pairs in relation to amino acid plasma profile and the effect sizes and the common language effect size statistic correspondent

| **Amino acids** | **FEP patients** | | **Siblings** | **FEP-Siblings pairs** | | | |
| --- | --- | --- | --- | --- | --- | --- | --- |
|  | **Mean (SD)** | | | **t-value** | **p-value** | **d (95% CI)** | **CL*** |
| GLU | 198.50 (128.22) | 170.11 (102.04) | | 0.19 | 0.852 | 0.70 (0.51, 0.89) | 76.0% |
| GLN | 339.46 (205.81) | 334.74 (280.07) | | -0.59 | 0.556 | 0.67 (0.46, 0.88) | 75.0% |
| GLY | 326.93 (131.41) | 330.60 (165.77) | | -0.29 | 0.773 | 0.60 (0.36, 0.84) | 73.0% |
| Glx | 537.96 (238.40) | 504.85 (276.83) | | -0.87 | 0.388 | 0.76 (0.61, 0.91) | 78.0% |
| GLN/GLU | 2.66 (3.03) | 3.41 (4.95) | | -0.52 | 0.607 | 0.61 (0.38, 0.84) | 73.0% |
| PRO | 347.14 (280.65) | 292.07 (170.35) | | -0.50 | 0.619 | 0.70 (0.51, 0.89) | 76.0% |
| SER | 153.94 (65.28) | 161.52 (69.99) | | -0.58 | 0.561 | 0.68 (0.48, 0.88) | 75.0% |
| TRP | 58.32 (21.49) | 62.78 (24.44) | | -1.72 | 0.091 | 0.66 (0.45, 0.87) | 75.0% |
| TYR | 82.30 (37.03) | 82.84 (35.51) | | -0.88 | 0.384 | 0.77 (0.62, 0.92) | 78.0% |
| GABA | 83.64 (29.82) | 90.58 (32.93) | | -1.56 | 0.124 | 0.58 (0.34, 0.82) | 72.0% |

Cohen´s d: Effect size index for means (small: 0.20-0.49, medium: 0.50-0.79; large: ≥0.80); CI: Confidence Interval; *CL: Common language effect size statistic.
